# Supplementary material for: Pan-cancer analysis reveals TREM1+ PMN-MDSCs as critical regulators of immune suppression and tumor microenvironment remodeling
Source: Commun Biol. 2025 Dec 18;9:75. doi: 10.1038/s42003-025-09342-8 (PMC12820143; doi:10.1038/s42003-025-09342-8)
Supplement: Supplementary file 2 — Supplementary Information [file 42003_2025_9342_MOESM2_ESM.pdf]

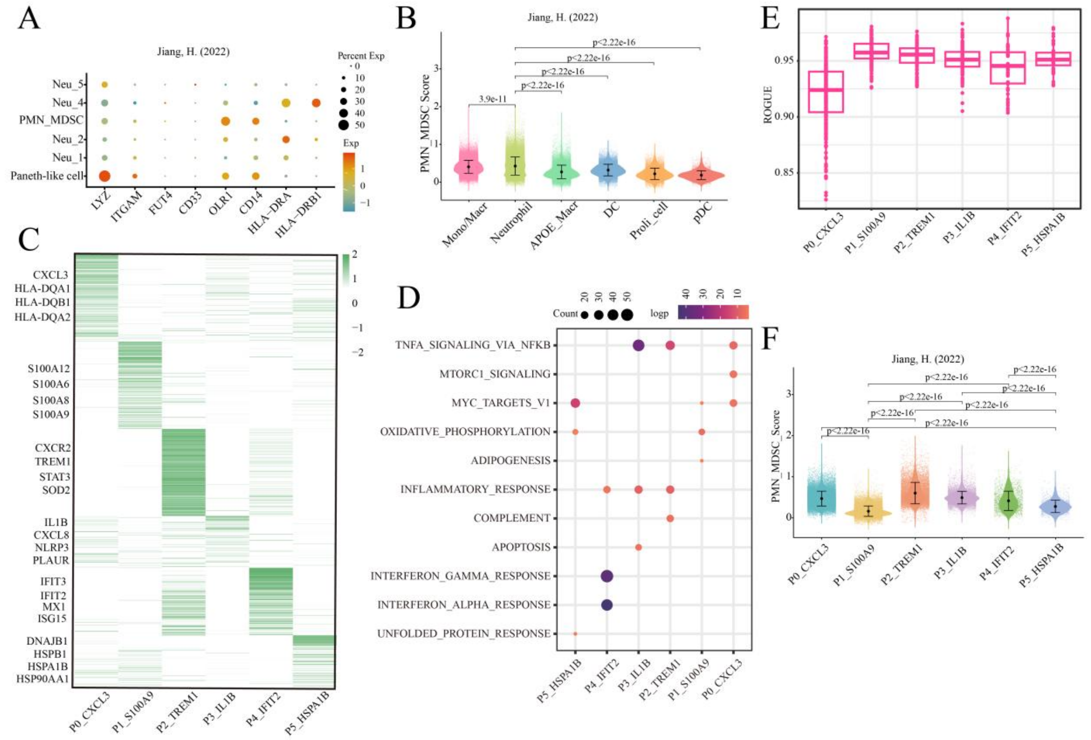

Supplementary Fig. 1. A. Bubble plot showing the marker of neutrophil clusters defined by scRNA-seq from Jiang et al. **B.** Violin plot showing the PMN-MDSC-specific gene set scores across myeloid subpopulations. **C.** Gene expression heatmap in neutrophil subsets. **D.** Enriched pathways in neutrophil subsets. **E.** ROGUE evaluation of neutrophil subset purity. **F.** Violin plot showing the PMN-MDSC-specific gene set scores across neutrophil subpopulations.

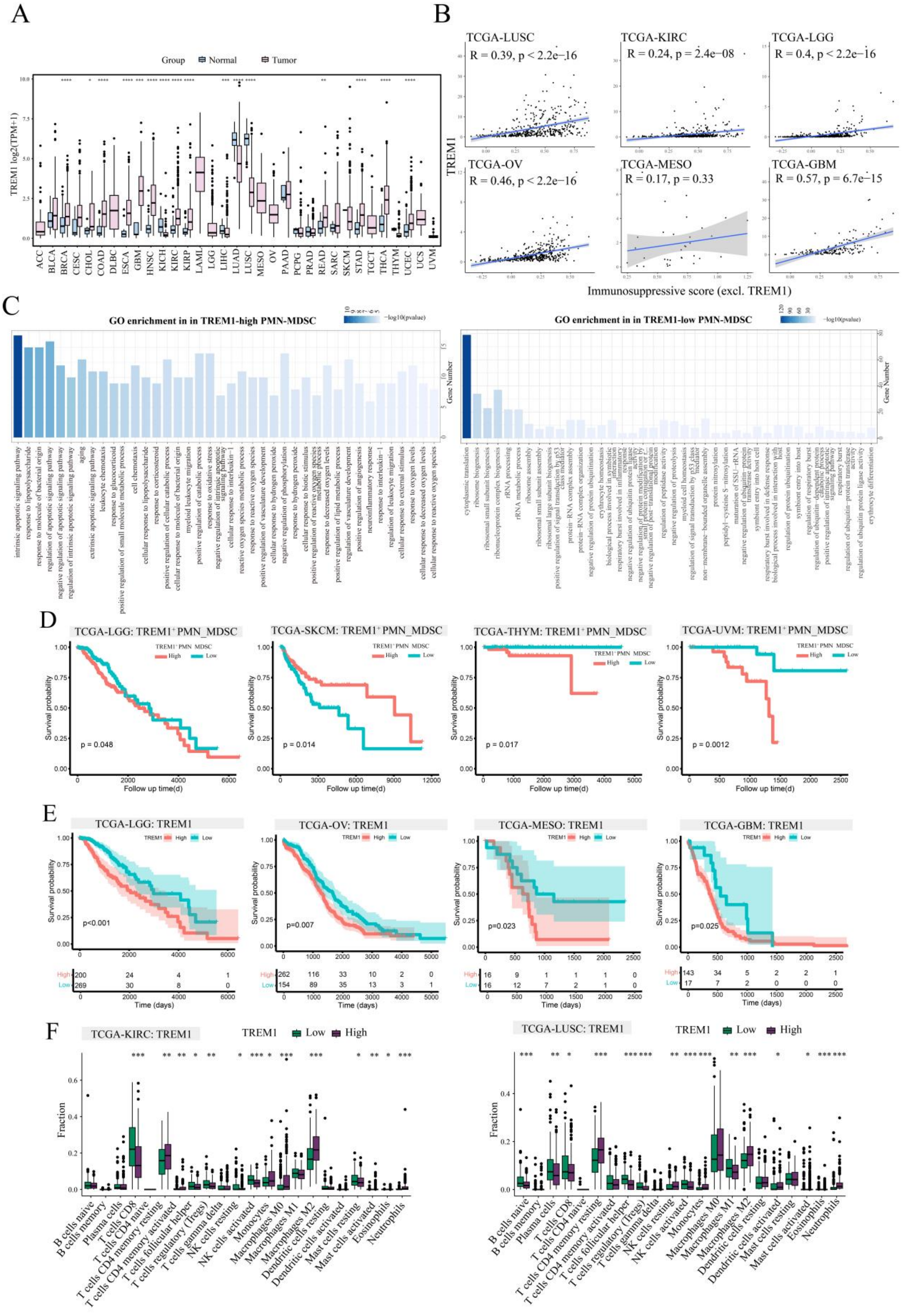

Supplementary Fig. 2. A. The box plotted by https://www.bioinformatics.com.cn showing the expression of *TREM1* across cancer types. **B.** The scatter plot showing the correlation between *TREM1* gene and immunosuppressive gene set (with *TREM1* removed) in a variety of cancer types. **C.** The bar graph showing the functional

enrichment of *TREM1*$^{+}$ high PMN-MDSC and *TREM1*$^{+}$ low PMN-MDSC. **D.** Prognostic analysis of *TREM1*$^{+}$ PMN-MDSCs subset across different cancer types. **E.** Prognostic analysis of the *TREM1* gene across different cancer types. **F.** Box plot showing the differences in the infiltration levels of immune cells between high and low *TREM1* gene infiltration. *$p < 0.05$, **$p < 0.01$, ***$p < 0.001$, ****$p < 0.0001$.

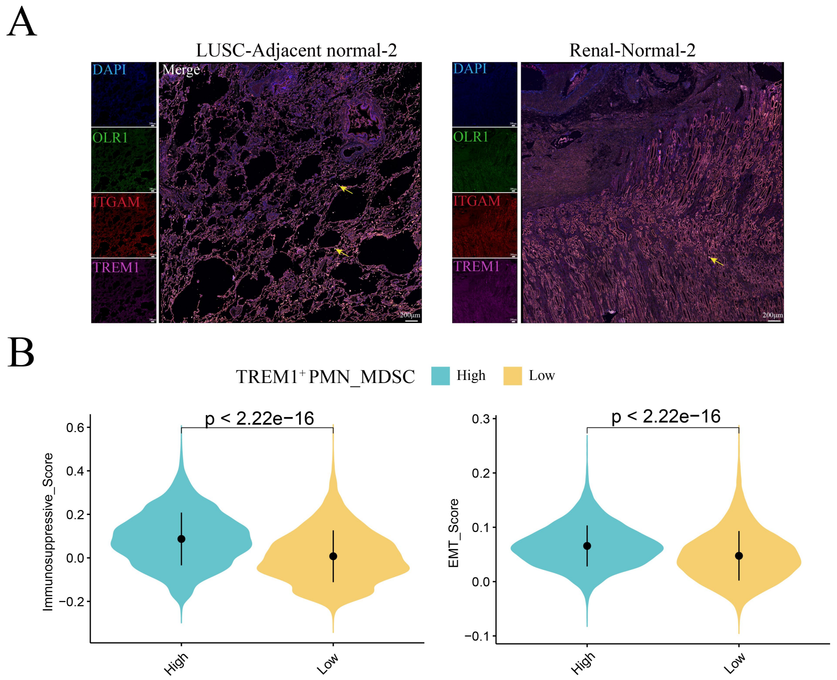

Supplementary Fig. 3. A. Multiplex immunofluorescence staining for *TREM1*$^{+}$ PMN-MDSCs in non-tumor tissue. DAPI (blue), *ITGAM* (red), *OLR1* (green) and *TREM1* (magenta) are shown in individual and merged channels. The yellow arrows point to cells positive for the three markers. **B.** Differences in the scores of immunosuppressive and EMT between high and low infiltration groups of *TREM1*$^{+}$ PMN-MDSCs.

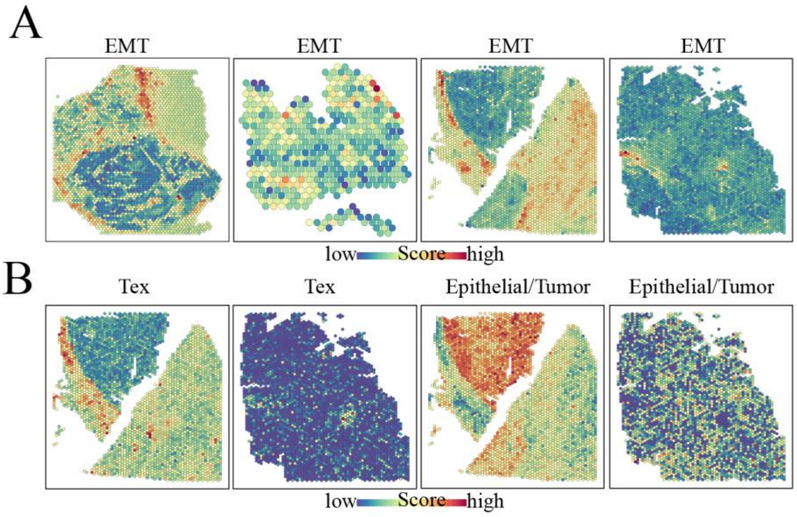

Supplementary Fig. 4. A. Spatial feature plots of EMT in tissue sections of BRCA, LC and KIRC. **B.** Spatial feature plots of exhausted T cells and epithelial/tumor cells in tissue sections of KIRC.

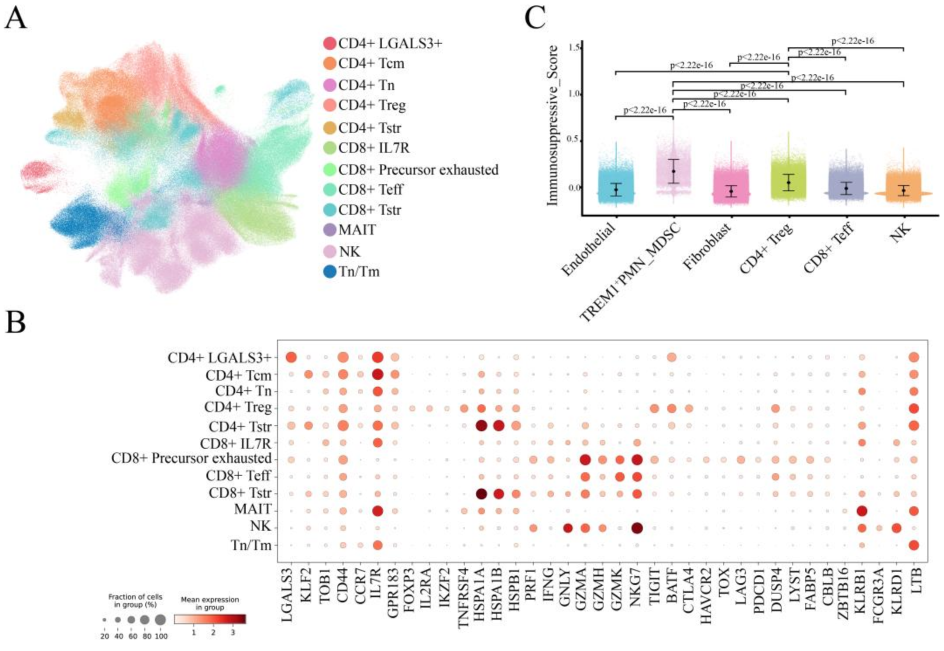

Supplementary Fig. 5. A. UMAP plot showing the distribution of T/NK cell subsets. **B.** Bubble plot displaying the markers of T/NK cell subsets. **C.** Violin plot showing the scores of immunosuppressive gene sets in different cell types.

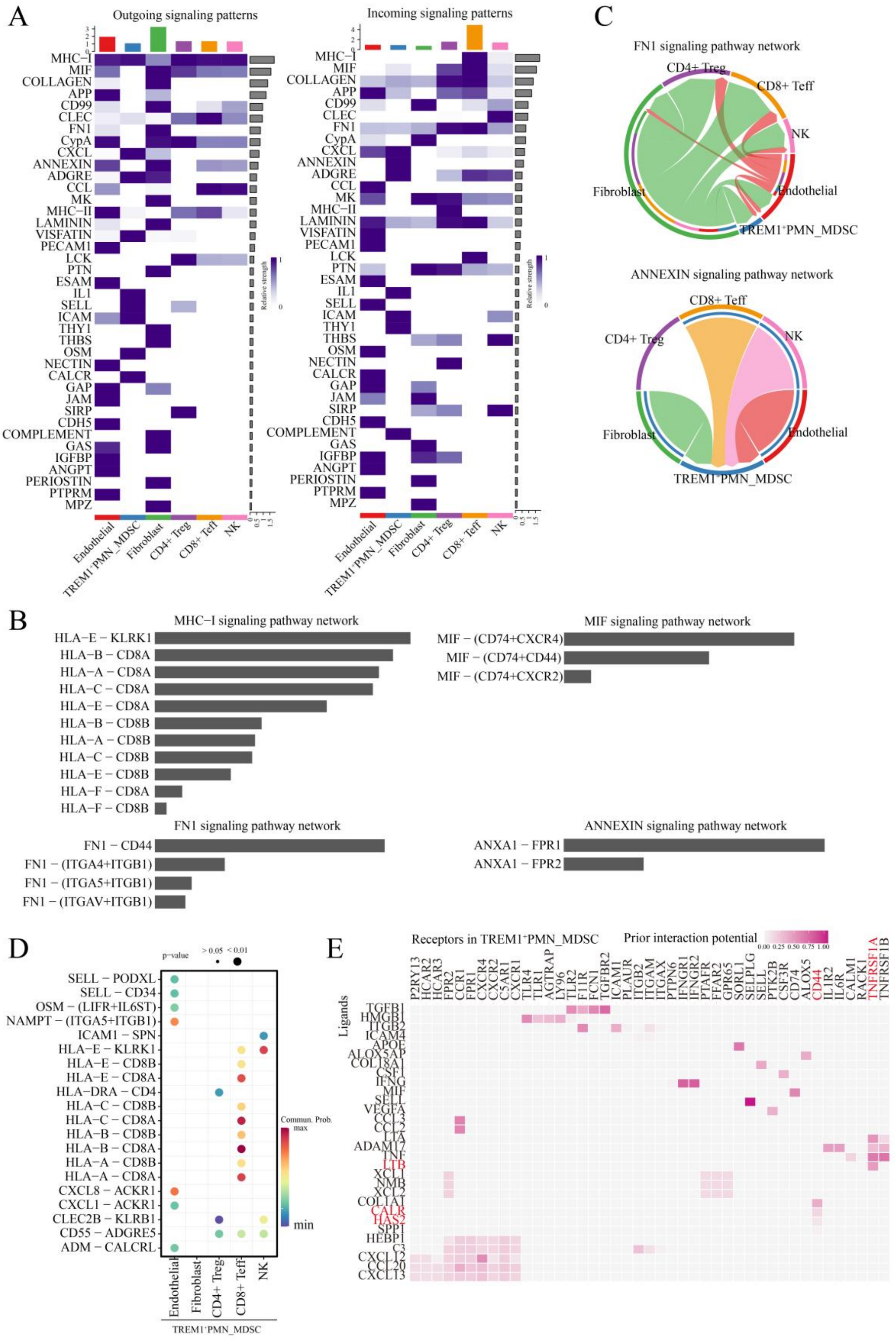

Supplementary Fig. 6. A. Heatmap showing the contribution of outgoing and incoming signaling pathways in different cell types. **B.** Bar plot showing the contribution of all L-R pairs in the respective pathways. **C.** Chord plot showing the FN1 and ANNEXIN signaling pathway networks. **D.** Bubble plot of L-R pairs from

TREM1$^+$ PMN-MDSCs to other interacting cells. **E.** Heatmap showing the L-R pairs between CD4$^+$ Treg, CD8$^+$ Teff, endothelial cell, fibroblast, NK cell and *TREM1*$^+$ PMN-MDSCs.
