# Supplementary material for: Pan-cancer analysis reveals TREM1+ PMN-MDSCs as critical regulators of immune suppression and tumor microenvironment remodeling
Source: Commun Biol. 2025 Dec 18;9:75. doi: 10.1038/s42003-025-09342-8 (PMC12820143; doi:10.1038/s42003-025-09342-8)
Supplement: Supplementary file 3 — Description of Additional Supplementary files [file 42003_2025_9342_MOESM3_ESM.pdf]

Description of Additional Supplementary Files

File name: Supplementary Data 1

Description: Supplementary material for scRNAseq datasets

File name: Supplementary Data 2

Description: Supplementary materials for the gene sets employed in bulk RNA-seq, scRNA-seq, and spatial transcriptomics

File name: Supplementary Data 3

Description: Supplementary materials for spatial transcriptomics datasets

File name: Supplementary Data 4

Description: Supplementary materials for cancer types and their abbreviations

File name: Supplementary Data 5

Description: Supplementary materials for functional signature gene sets

File name: Supplementary Data 6

Description: Supplementary material for CCK8 assays

File name: Supplementary Data 7

Description: Supplementary material for ELISA assays
