# Supplementary material for: Pan-cancer analysis reveals TREM1+ PMN-MDSCs as critical regulators of immune suppression and tumor microenvironment remodeling
Source: Commun Biol. 2025 Dec 18;9:75. doi: 10.1038/s42003-025-09342-8 (PMC12820143; doi:10.1038/s42003-025-09342-8)
Supplement: Supplementary file 5 — Reporting Summary [file 42003_2025_9342_MOESM5_ESM.pdf]

nature portfolio

Corresponding author(s): COMMSBIO-25-3020A

Last updated by author(s): Nov 16, 2025

Reporting Summary

Nature Portfolio wishes to improve the reproducibility of the work that we publish. This form provides structure for consistency and transparency in reporting. For further information on Nature Portfolio policies, see our Editorial Policies and the Editorial Policy Checklist.

Statistics

For all statistical analyses, confirm that the following items are present in the figure legend, table legend, main text, or Methods section.

n/a	Confirmed	
☐	☒	The exact sample size (*n*) for each experimental group/condition, given as a discrete number and unit of measurement
☐	☒	A statement on whether measurements were taken from distinct samples or whether the same sample was measured repeatedly
☐	☒	The statistical test(s) used AND whether they are one- or two-sided<br>*Only common tests should be described solely by name; describe more complex techniques in the Methods section.*
☒	☐	A description of all covariates tested
☒	☐	A description of any assumptions or corrections, such as tests of normality and adjustment for multiple comparisons
☒	☐	A full description of the statistical parameters including central tendency (e.g. means) or other basic estimates (e.g. regression coefficient) AND variation (e.g. standard deviation) or associated estimates of uncertainty (e.g. confidence intervals)
☒	☐	For null hypothesis testing, the test statistic (e.g. *F*, *t*, *r*) with confidence intervals, effect sizes, degrees of freedom and *P* value noted<br>*Give P values as exact values whenever suitable.*
☒	☐	For Bayesian analysis, information on the choice of priors and Markov chain Monte Carlo settings
☒	☐	For hierarchical and complex designs, identification of the appropriate level for tests and full reporting of outcomes
☒	☐	Estimates of effect sizes (e.g. Cohen's *d*, Pearson's *r*), indicating how they were calculated

Our web collection on statistics for biologists contains articles on many of the points above.

Software and code

Policy information about availability of computer code

Data collection	Data used in the study were downloaded from public database, detailed information has been provided in the Supplementary Data.
Data analysis	All analyses were performed using R software and Python3.9. The code script utilized for analysis in this study can be accessed at https://github.com/haijun2595/MDSC. The following packages were used: Seurat(4.1.0),Scanpy(v.1.10.1), scProgram(v0.0.0.9), GSVA(v1.42.0), clusterProfiler(v4.2.2), pySCENIC(v0.12.1), survival(v3.2-13), survminer(v0.5.0), CellChat(v1.6.1), BayesSpace(v1.15.3), mistyR(v1.10.0), stLearn(v0.4.12), nichenetr(v2.1.5). Statistical analyses were performed using GraphPad Prism 9.5 and R software.

For manuscripts utilizing custom algorithms or software that are central to the research but not yet described in published literature, software must be made available to editors and reviewers. We strongly encourage code deposition in a community repository (e.g. GitHub). See the Nature Portfolio guidelines for submitting code & software for further information.

Data

Policy information about availability of data

All manuscripts must include a data availability statement. This statement should provide the following information, where applicable:

- Accession codes, unique identifiers, or web links for publicly available datasets
- A description of any restrictions on data availability
- For clinical datasets or third party data, please ensure that the statement adheres to our policy

Provide your data availability statement here.

Research involving human participants, their data, or biological material

Policy information about studies with human participants or human data. See also policy information about sex, gender (identity/presentation), and sexual orientation and race, ethnicity and racism.

Reporting on sex and gender	Our study did not include RNA-seq data analysis on sex differences.
Reporting on race, ethnicity, or other socially relevant groupings	No socially constructed or socially relevant categorization variable used in this study.
Population characteristics	Data used in the study were downloaded from public database. The study participants included healthy individuals� cancer patients� and patients who non-neoplastic disease.
Recruitment	No patient recruitment involved, as this is not a prospective study. This is not a clinical trial.
Ethics oversight	The acquisition of samples and tissue sections was approved by the Ethics Committee of the First Affiliated Hospital of Guangxi Medical University (approval number: KY-E-097) and The Fifth Affiliated Hospital of Guangxi Medical University (LW2024-DECISION-010). The collection of peripheral blood samples from healthy individuals and spleen samples from mice was apprlineoved by the Ethics Committee of the First Affiliated Hospital of Guangxi Medical University (approval number: 2025-E0787). The patients/participants were properly informed and provided their written informed consent to participate in this study. This study was conducted in accordance with the Declaration of Helsinki. All ethical regulations relevant to human research participants were followed.

Note that full information on the approval of the study protocol must also be provided in the manuscript.

Field-specific reporting

Please select the one below that is the best fit for your research. If you are not sure, read the appropriate sections before making your selection.

☒ Life sciences ☐ Behavioural & social sciences ☐ Ecological, evolutionary & environmental sciences

For a reference copy of the document with all sections, see nature.com/documents/nr-reporting-summary-flat.pdf

Life sciences study design

All studies must disclose on these points even when the disclosure is negative.

Sample size	We analyzed a total of 576 clinical samples comprising 2,565,798 cells, with complementary bulk RNA-seq expression data from 29 cancer types obtained from TCGA database.
Data exclusions	No data were excluded from the analyses.
Replication	The reproducibility of all analyses was confirmed.
Randomization	The samples in this study were obtained from patients' tissues, with outcomes that had already occurred at the time of their admission. No intervention measures or group allocations were set afterward. Additionally, this study integrated data published by other researchers, making randomization not applicable to our research.
Blinding	Blinding was not applicable in this study.

Reporting for specific materials, systems and methods

We require information from authors about some types of materials, experimental systems and methods used in many studies. Here, indicate whether each material, system or method listed is relevant to your study. If you are not sure if a list item applies to your research, read the appropriate section before selecting a response.

Materials & experimental systems

n/a	Involved in the study
☐	☒ Antibodies
☐	☒ Eukaryotic cell lines
☒	☐ Palaeontology and archaeology
☐	☒ Animals and other organisms
☒	☐ Clinical data
☒	☐ Dual use research of concern
☒	☐ Plants

Methods

n/a	Involved in the study
☒	☐ ChIP-seq
☒	☐ Flow cytometry
☒	☐ MRI-based neuroimaging

Antibodies

Antibodies used	Anti-CD11b antibody (Abcam, ab52478, 1:400 dilution), Rabbit Anti-LOX1 antibody (Bioss, bs-2044R, 1:400 dilution), and Rabbit Anti-TREM1 antibody (Bioss, bs-10306R, 1:400 dilution). Immunocomplexes were detected using Alexa Fluor-conjugated secondary antibodies (Life Technologies, 1:400 dilution).
Validation	All the antibodies have been validated for immunofluorescence staining on human tissue sections both on the manufacturer's website and by our own experimental data.

Eukaryotic cell lines

Policy information about cell lines and Sex and Gender in Research

Cell line source(s)	The human renal cell carcinoma cell line 786-O was purchased from Immocell,and the human lung adenocarcinoma cell line H226 was purchased from Procell Company. T cells were isolated from healthy volunteer's peripheral blood. MDSCs were isolated from the spleens of healthy C57BL/6J mice with the MDSC isolation kit.
Authentication	The 786-O and H226 cell lines were purchased from Immocell and Procell Company, respectively, and were not further authenticated. T cells and MDSCs were isolated using commercial kits and were not further authenticated.
Mycoplasma contamination	All cell lines tested negative for mycoplasma contamination.
Commonly misidentified lines (See ICLAC register)	No commonly misidentified cell lines were used in the study.

Animals and other research organisms

Policy information about studies involving animals; ARRIVE guidelines recommended for reporting animal research, and Sex and Gender in Research

Laboratory animals	Female C57BL/6J mice (n=5), aged 6-8 weeks were obatained from GuangXi Medical University Animal Center.
Wild animals	The study did not involve wild animals.
Reporting on sex	This study included only female C57BL/6J mice.
Field-collected samples	The study did not involve samples collected from the field.
Ethics oversight	The Ethics Committee of the First Affiliated Hospital of Guangxi Medical University approval number:2025-E0787 .

Note that full information on the approval of the study protocol must also be provided in the manuscript.

Plants

Seed stocks	Not appliable.
Novel plant genotypes	Not appliable.
Authentication	Not appliable.
